# Targeting Mitochondrial Biogenesis with Polyphenol Compounds

**DOI:** 10.1155/2021/4946711

**Published:** 2021-07-12

**Authors:** Leila Chodari, Mutlu Dilsiz Aytemir, Parviz Vahedi, Mahdieh Alipour, Sepideh Zununi Vahed, Seyed Mahdi Hosseiniyan Khatibi, Elham Ahmadian, Mohammadreza Ardalan, Aziz Eftekhari

**Affiliations:** ^1^Physiology Department, Faculty of Medicine, Urmia University of Medical Sciences, Urmia 571478334, Iran; ^2^Hacettepe University, Faculty of Pharmacy, Department of Pharmaceutical Chemistry, 06100, Sıhhiye, Ankara, Turkey; ^3^İzmir Katip Çelebi University, Faculty of Pharmacy, Department of Pharmaceutical Chemistry, 35620, Çiğli, İzmir, Turkey; ^4^Department of Anatomical Sciences, Maragheh University of Medical Sciences, Maragheh, Iran; ^5^Dental and Periodontal Research Center, Faculty of Dentistry, Tabriz University of Medical Sciences, Tabriz, Iran; ^6^Kidney Research Center, Tabriz University of Medical Sciences, Tabriz, Iran; ^7^Pharmacology and Toxicology Department, Maragheh University of Medical Sciences, Maragheh, Iran

## Abstract

Appropriate mitochondrial physiology is an essential for health and survival. Cells have developed unique mechanisms to adapt to stress circumstances and changes in metabolic demands, by meditating mitochondrial function and number. In this context, sufficient mitochondrial biogenesis is necessary for efficient cell function and haemostasis, which is dependent on the regulation of ATP generation and maintenance of mitochondrial DNA (mtDNA). These procedures play a primary role in the processes of inflammation, aging, cancer, metabolic diseases, and neurodegeneration. Polyphenols have been considered as the main components of plants, fruits, and natural extracts with proven therapeutic effects during the time. These components regulate the intracellular pathways of mitochondrial biogenesis. Therefore, the current review is aimed at representing an updated review which determines the effects of different natural polyphenol compounds from various plant kingdoms on modulating signaling pathways of mitochondrial biogenesis that could be a promising alternative for the treatment of several disorders.

## 1. Introduction

Healthy physiology of mitochondria is essential for human health, and their dysfunction is connected with the pathogenesis of different diseases such as type 2 diabetes, cancer, neurodegenerative, and cardiovascular diseases [1–5]. For this reason, the removal of damaged mitochondria (mitophagy) and the generation of new ones are required to preserve the cellular and mitochondrial homeostasis.

Emerging evidence indicates that the biogenesis and clearance of mitochondria are tightly controlled by a multistep process involving fusion, fission, and mitophagy events [6]. The biogenesis of mitochondria is extremely plastic in response to environmental stimuli, developmental signals, and cellular energy demand [7] and requires mitochondrial DNA (mtDNA) replication, synchronous production of mitochondrial and nuclear proteins, and localization of nuclear-encoded proteins and phospholipids in different subcompartments of mitochondria [8, 9].

Recent data highlight the impact of the peroxisome proliferator-activated receptor-*γ* coactivator- (PGC-) 1*α*, a master controller of mitochondrial biogenesis. PGC-1*α*, through activation of different transcription factors, mainly Nrf-1 (nuclear respiratory factor 1) and Nrf-2 triggering mitochondrial transcription factor A (TFAM), promotes mitochondrial biogenesis [8, 10]. Nrf-2 also induces mtDNA replication and transcription. The activation of the PGC-1*α*/Nrf/TFAM pathway results in mtDNA and protein synthesis and generation of new mitochondria [11, 12]. Different key factors including ROS production, nitric oxide, calcineurin, AMPK (AMP-activated protein kinase), energy sensor, and sirtuins can induce PGC-1*α* and mitochondrial biogenesis [8, 13].

Given the essential role of mitochondrial impairment in the pathogenesis of different diseases, the knowledge of the molecular mechanisms, signals, and actors regulating the biogenesis and function of mitochondria is thus of high prominence which can be used to develop novel mitochondrial-based therapies. Numerous natural extracts such as polyphenols, flavonoids, and saponins are reported to antioxidant and anti-inflammatory activities [14, 15] and stimulate the biogenesis of mitochondria. In this review, we will highlight an updated report of implying the effects of polyphenol compounds on mitochondrial biogenesis and function by modulating intracellular signaling pathways in different models focusing on biogenesis processes as the main mechanism involved in the modulation of energy expenditure rather than their well-known antioxidant properties.

## 2. Mitochondrial Biogenesis

The membrane-enclosed organelles recognized as “power generators” of eukaryotic cells are called mitochondria. Moreover, various metabolic pathways (Krebs cycle, OXPHOS (oxidative phosphorylation), pyruvate oxidation, fatty acid beta-oxidation, etc.) are carried out through their molecular cascades [5]. Mitochondria's advantages are not only for their contribution to cell metabolism but also are required to regulate energy-producing pathways. Their commitment to steering stress responses and cell death has gained significant attention lately [16–18]. Additionally, inside the cells, mitochondria serve as the ROS's prime source [19]. Mitochondria's biomedical growing concerns are due to their diverse executions covering multiple genetic diseases (through inheriting mitochondrial DNA (mtDNA) mutations), contributions to neurodegeneration, aging, inflammation, and carcinogenesis [20].

The biogenesis of organelles is connected with their inheritance through cellular division. Mitochondrial biogenesis is linked with the proliferation and division of prior organelles, a process which is coordinated by cell cycle cascades [21]. However, different stimuli such as exercise, oxidative stimulus, elevated energy demand, hormones, and physiological development as well as certain diseases also trigger mitochondrial biogenesis [22]. The individual mitochondrial mass increases during mitochondrial biogenesis. Mitochondrial biogenesis has recently been proposed as a potential target of intervention in different diseases which have not an effective cure up to date. This is due to the crucial roles of mitochondria in numerous cellular events [23]. Thus, pharmacological manipulations are plausible in order to induce mitochondrial biogenesis.

Besides proceeding mitochondrial biogenesis among healthy cells, the importance of mitochondrial biogenesis and oxidative phosphorylation in damaged cells has been reported [24]. Stimulation of PGC-1*α* (through deacetylation or phosphorylation) enrols the pathway, which is later followed by stimulation of nuclear transcription series factors such as estrogen-related receptor-*α* (ERR-*α*), NRF-1, and NRF-2, and succeeding with a rise in TFAM's expression ultimately leads to mtDNA transcription and replication [22, 25, 26]. The translation of mtDNA encoded genes is facilitated by particular translation factors, including the initiation factor 2 (mtIF2) and mtIF3, the elongation factors Tu (mtEFTu), mtEFT, and mtEFG1, and translational release factor 1-like (mtRF1L) and recycling factors 1 (mtRRF1) and mtRRF2. Besides, the binding of mitochondrial RNA (mRNA) to the translational activator of cytochrome c oxidase 1, aka TACO1, controls the mitochondrial proteins' level.

**Figure 1:**
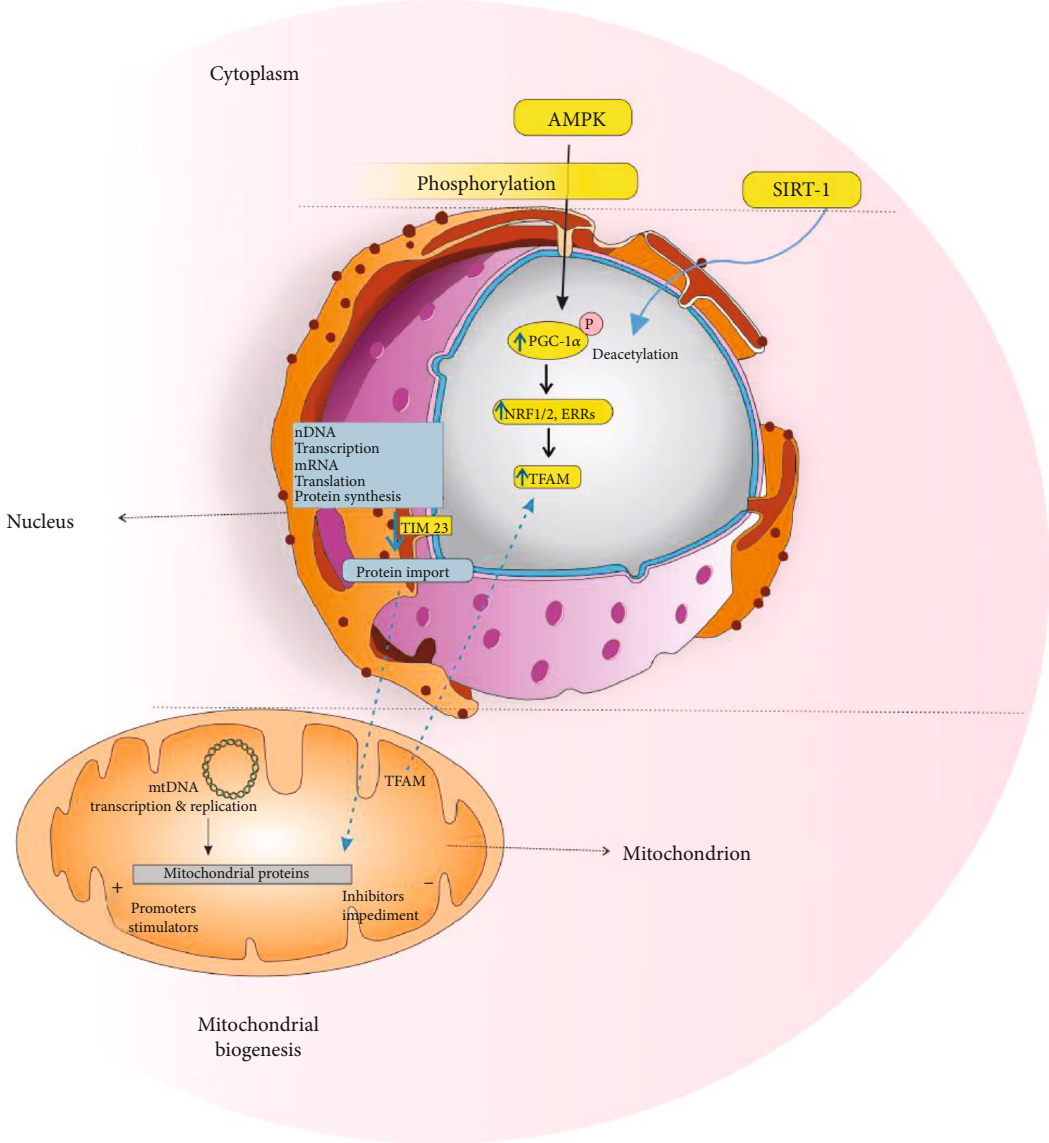
The role of different signaling pathways in mitochondrial biogenesis.

Triggering of silent information regulator-1 (SIRT1) initiates transcription of nuclear and mitochondrial genes encoding proteins mediated by PGC-1*α*, which occurs during the proliferation of mitochondria. The amplified production of proteins contributed to the oxidation of fatty acid, oxidative phosphorylation, and tricarboxylic acid cycle in handled by SIRT3. Additionally, in parallel to that, AMPK and PGC-1*α* are affected collaterally. C1q/tumor necrosis factor-related protein-3 contributes to the AMPK/PGC-1*α* pathway that advances biogenesis. Additionally, the phosphorylation and activation of histone acetyltransferase 1 by AMPK lead to a far less constricted chromatin-DNA structure that ultimately activates transcription. Besides, phosphorylation of DNA methyltransferase 1 as an epigenetic factor, aka DNMT1 by AMPK, restricts the access of transcription factors to promoters. [27].  Figure 1 represents the signaling pathways involved in mitochondrial biogenesis.

Synthesis, importing, and assembling of nDNA-encoded mitochondrial proteins are important orchestrated steps. Cytosolic preproteins are the source of such mitochondrial proteins and compromise targeting signals at amino-terminals. Translocase TIM23 transmits the messages of these preproteins to the mitochondrial matrix. Within there, they are assembled and assorted to the matrix of the inner mitochondrial membrane (IMM). This pathway's energy supply is catered through oxidative phosphorylation (ATP) and the mitochondrial membrane potential [28, 29]. Conducted studies of outer mitochondrial membrane (OMM)'s biogenesis have been done on yeast, Saccharomyces cerevisiae, and other unicellular organisms. The OMM plays an essential role in communication with other intracellular organelles and is crucial for fission, fusion, and other processes contributing to mitochondrial dynamic changes. [30]. The escalation of mtDNA:nDNA percentage, the expression level of the mitochondrial gene, and mtDNA quantity indicate the level of mitochondrial biogenesis [31]. Although cancerous cells have declined mitochondria number, some extracted data present increments in the expression of TFAM, PGC-1*α*, and NRF1 genes [32–34]. However, a late report raises the uncertainty of the TFAM level's indicated as a biogenesis marker, due to its failure in accommodation to mtDNA quantity and the expression of mtDNA-encoded polypeptides [35]. The consequences of mitochondrial biogenesis comprise of boosting of mitochondrial-associated dysfunction, declining of pathologic oxidative stress, and oxidative phosphorylation efficiency. [25]. mtDNA synthesis and mitochondrial membrane phospholipids are encompassed with certain strategies for measuring mitochondrial biogenesis. Notably, variation in mitochondria's number do not represent a definite sign of biogenesis (because number changes are not limited to synthesis) [36]. Moreover, remarkably, mitochondrial biogenesis could lead to detrimental effects, including reactions of unfolded protein's silencing to the endoplasmic reticulum and misfolded proteins' organelle importation [37].

## 3. Natural Agents Modifying Mitochondrial Biogenesis

Within the physiological circumstances, cells react to diminished energy by affecting transcription factors through up-/downregulation, which contributes to mitochondrial biogenesis enhancement and/or restraining (Table 1). Pathological disturbances to mitochondrial biogenesis lead to two conditions: (a) hampering, this requires stimulating of the process, and (b) abnormal intensifying. Enhancing mitochondrial biogenesis is up to transcription factors' activation that affects stimulating local translation of mitochondrial proteins and mitochondrial genes. Polyphenols have been considered as the main natural compounds interfering with mitochondrial biogenesis. Thus, the present review focuses on the role of these components in the regulation of mitochondrial biogenesis.

## 4. Polyphenols

Activating mitochondrial biogenesis in vitro has been indicated by various polyphenols. Hence, they are under inspection as potential inducers through initiating PGC-1*α* activation by deacetylation as a mediator [10].

### 4.1. Phenolic Compounds

#### 4.1.1. Resveratrol

At the point, resveratrol appears to stimulate PGC-1*α* activity through boosting deacetylation with SIRT1's mediation and enhancing transcriptional activity in the liver and muscles tissues of mice [38, 39]. Additionally, resveratrol promotes the motor function and survival rate of high fat diet-received mice [38, 39]. In a recently published paper, the effects of resveratrol on the activation of PGC-1*α*/SIRT1 cascade have shown an upward trend in endothelial cells *in vitro* [40]. Besides, similar effects were observed in the aorta of type 2 diabetes (T2D) animals and the cardiac tissues of human renin angiotensin genes expressing transgenic rats [41]. Despite these beneficial effects, the exact molecular mechanisms of resveratrol-induced mitochondrial biogenesis remain unclear. Resveratrol's effects on mediating mitochondrial biogenesis through initiating AMP protein kinase (AMPK) have been presented by various authors [42]. Indeed, evidence illuminating the blockade of oxidative phosphorylation (OXPHOS) complex III, phosphodiesterase, or ATP synthase by resveratrol is mainly contributed to the activation of AMPK [43–46]. Moreover, extending the levels of intracellular NAD+ due to triggering of SIRT1 as a collateral outcome of resveratrol's contribution to AMPK has been suggested [47]. Other researches have proposed that resveratrol activates SIRT1, which in turn terminates in liver kinase B1 deacetylation and AMPK activation accompanied by enhancement (in vivo and vitro) of mitochondrial function [47–50]. Besides, the direct involvement of resveratrol in OXPHOS system has been detected. Resveratrol influences the activity of mitochondrial complex I via binding to the subunits of nicotinamide adenine dinucleotide (NADH) dehydrogenase, which contributes to an antagonistic characteristic on OXPHOS, in which low dose resveratrol stimulates complex I activity and high dose results in its prohibition in liver cells [51]. Resveratrol substantially enhances the function of complex I in young mice, while old animals are not influenced [52]. Throughout another experiment, resveratrol was shown to inhibit the function of complex III through competing with coenzyme Q *in vitro* [53]. Conversely, cellular respiratory capacity and the activity of complexes I to IV have shown a surge in a recent *in vitro* study subsequent to resveratrol treatment [54]. It has been revealed that resveratrol shows regulatory effects on the synthesis of ATP and complex V, while in lower doses stimulates the complex V activity [55] and derives an inhibiting role on the generation of ATP [56]. Recently, the role of resveratrol in the maintenance of cellular mitochondrial DNA replication was assessed. Among human fibroblasts derived from (skin of) patients carrying homoplasmic mutations of the mitochondria-encoded ATP Synthase Membrane Subunit 6 (MT-ATP6), mitochondria-encoded TRNA Leucine 1 (UUA/G) (MT-TL1), and mitochondrial-encoded TRNA Lysine (MT-TK) gene, resveratrol excessed primary oxygen intake rates and ATP formation [57]. Besides, resveratrol provokes fusion in mitochondria, leading to extremely expanded mitochondrial network divisions. Additionally, in patients suffering from Complex I and IV inadequacy, resveratrol has demonstrated growth in fibroblasts' biogenesis of mitochondria [58]. Correspondingly, in isolated cells from patients suffering from complex I inadequacy consuming resveratrol, the ratios of lactate to pyruvate became normal. Additionally, it regained the ratios of NADH to NAD+. Serious lactic acidosis develops among patients with a deficiency of OXPHOS [59]. This reassures the previous finding [44], and resveratrol consumption can have advantageous gains in such conditions. Resveratrol treating was promising among fibroblasts of patients suffering from complex inadequacy. The authors discovered a considerable ROS level plunge and increasing in SOD2 mediated by growth in SIRT3 activity [60]. Therefore, current evidence indicates the beneficial effects of resveratrol in OXPHOS-related diseases.

The effects of resveratrol on mitochondrial biogenesis has been investigated and compared with the flavonoids quercetin, catechin, berberine (a quaternary alkaloid and O-methyl polyphenol impacting mitochondria), cyanidin (various forms of 3′,4′-dihydroxyflavonoids), and cyanidin-3-glucoside (an intactly absorbed representative glycoside). After exposure (46% after 24 hours and 30% after 72 hours) to benzo[a]pyrene (B[a]P), the membrane potential of mitochondria (MMP) and cellular ATP was declined, and production of cellular ROS and superoxide in mitochondria was elevated. Reduced mitochondrial content could be preserved by polyphenol content after 72 h by 75%. The 24 h mRNA measurements of the activities of SIRT1 and other related proteins in mitochondria proposed interference with the biogenesis of mitochondria by the impact of this polyphenols. Furthermore, combined treatment with polyphenols mitigated mitochondrial deficiencies due to benzo[a]pyrene (primarily resveratrol) and developed the production of ROS and mitochondrial superoxide. Remarkably, 24 hours polyphenol pretreatment severely suppressed further B[a]P-stimulated increases in the production of ROS and mitochondrial superoxide, in which resveratrol is the most effective agent. Furthermore, the evidence promotes resveratrol as a chemoprophylaxis for enhancing mitochondrial biogenesis [61]. Resveratrol has also shown beneficial effects in several in vitro and in vivo models of neuropathies such as Alzheimer's, Down syndrome, cognition impairments, and aging via modulation of SIRT1-AMPK-PGC1-*α* pathway [62].

#### 4.1.2. Quercetin

**Figure 2:**
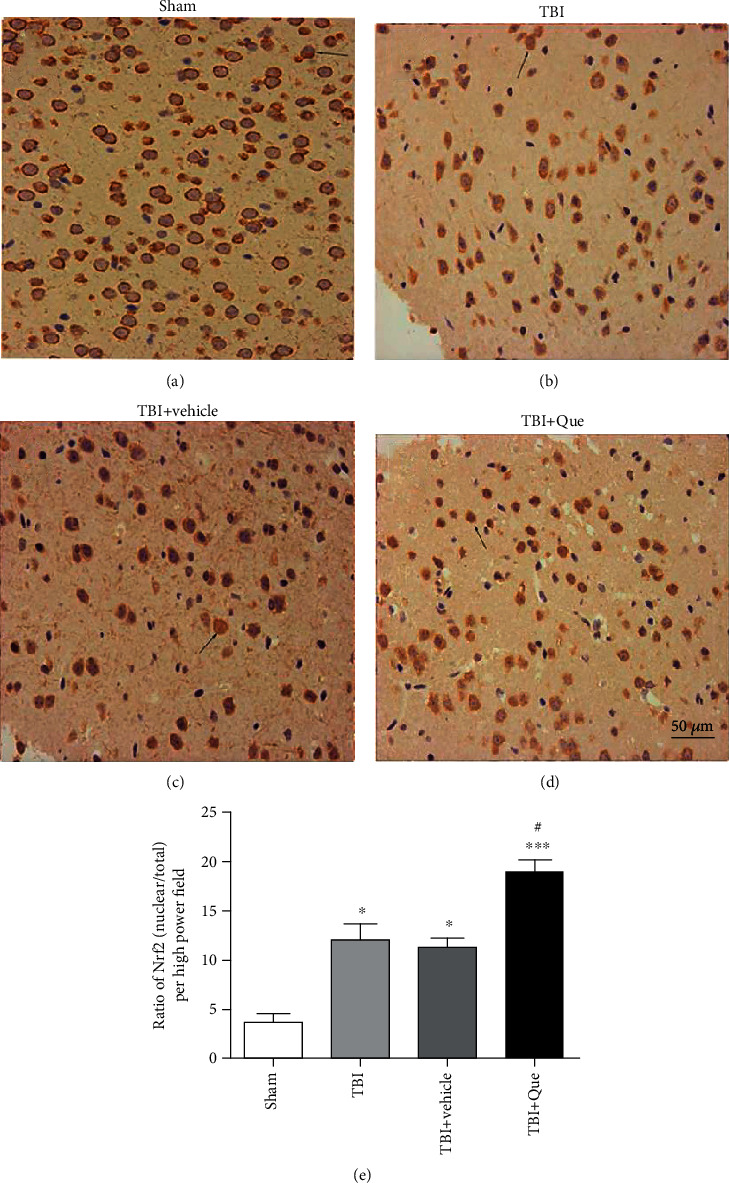
Immunohistochemistry and ratio of Nrf2 in mouse brain 24 h after traumatic brain injury in different groups ((a) sham, (b) traumatic brain injury, (c) traumatic brain injury with vehicle, (d) effect of quercetin on the concentration of Nrf2 in the nucleus, and (e) significant increase in the ratio of Nrf2 in the quercetin group in compared with the traumatic brain injury/vehicle group). Data represent the mean ± SEM (*n* = 5 per group). Scale (20 *μ*m). Reproduced under the terms and conditions of the Creative Commons Attribution 4.0 International License. Copyright 2016, PLOS [67].

Quercetin as another polyphenol has been excessively investigated and has displayed significant efficacy in inducing mitochondrial biogenesis. In the brain and skeletal muscle cells of mice, reports demonstrate induction in cytochrome C content, triggering SIRT1 and PGC-1*α*, and the mitochondrial DNA [63]. A concurrent physical endurance development in quercetin-administered animals has been reported. Similar practical impact engaged in enhancing the PGC-1*α* and SIRT1 expression [63]. A study on young adult male individuals (untrained) assessed the impact of quercetin on the biogenesis of mitochondria along with endurance exercise. Investigations showed that quercetin improved mtDNA number besides the notable increase in physical performance [64]. In line, in vitro findings proposed that administration of quercetin elevated the mitochondrial DNA content [65] and in a dose-dependent manner affected the expression levels of TFAM, NRF-1, and PGC-1*α*. Moreover, the OXPHOS complex IV function was also enhanced with quercetin. Hence, the collected data from experimental investigations suggest that quercetin, by assisting PGC-1*α*/NRF-1 and TFAM signaling, stimulates biogenesis of mitochondria [66]. Furthermore, the involvement of quercetin in the upregulation of Nrf2 expression and cytochrome c affects the mitochondrial function in traumatic brain injury in male mice (Figure 2) [67]. Quercetin also has elevated mitochondrial DNA levels and contents of TFAM, PGC-1*α*, and NRF-1 in a dose-depending way in the rat hippocampus under hypobaric hypoxia [67, 68]. Additionally, quercetin improves complexes II, IV, and V functions, besides ATP level's growth, therefore influencing the activity of OXPHOS [68]. Similar consequences were derived from the striatum and hippocampus exposed to aluminum of the rodent. Here, through the PGC-1*α*/NRF-1-NRF-2-TFAM cascade, quercetin provokes the biogenesis of mitochondria and maintains the mitochondrial shape and number [69].

Rayamajhi et al. perceived that 15 *μ*M quercetin provoked mtDNA quantity after 1 hour in a cultured human hepatocellular carcinoma cell line (HepG2 cells) [65]. Quercetin simultaneously leads to the upregulation of TFAM, PGC-1*α*, and NRF-1 in a dose- and time-dependent manner. Furthermore, the authors indicated that quercetin surged the mETC constituent of complex IV in the respiratory chain in immunohistochemistry studies. The data derived from research concerning the process of quercetin eliciting impacts manifest amplified genes associated with the biogenesis of mitochondrial genes in a heme oxygenase-1- (HO-1-) dependent way. Quercetin augmented the mRNA of heme oxygenase-1 and immune-ocontent across 0.5–3 hours. Additionally, quercetin impacts on complex IV, NRF-1, mitochondrial DNA, TFAM, and PGC-1*α* were restrained by combined treatment with an HO-1 suppressor, tin protoporphyrin IX (SnPP). Moreover, exposure of quercetin-treated HepG2 cells to hemoglobin (CO scavenger) terminated in resembled outcomes, including dropped mtDNA and the quantity of complex IV, and plummeted expression of molecules attributed to mitochondrial biogenesis, when contrasted to HepG2 cells having SnPP treatment. These researchers further discovered (in vivo) stimulated mouse liver mitochondrial biogenesis through HO-1/CO/PGC-1*α*/NRF-1-TFAM-dependent fashion occur when quercetin (50 mg/kg) supplementations are added for 7 days. Correspondingly, Kim et al. [70] lately inspected the expression of PPAR*α* and PGC-1*α* through an Nrf-2/HO-1-dependent process in primary hepatocytes of animals when receiving 10 *μ*M quercetin for 24 hours. Hence, carbon monoxide uses especial suppressors to afflict the impact of quercetin on the biogenesis of mitochondria. Therefore, quercetin appears to have an essential effect on the enhancement of mitochondrial biogenesis. However, in stimulating mitochondrial biogenesis in primary cortical neurons facing diminished oxygen and glucose, quercetin (0.1 or 1 *μ*M for 24 h) fails to show significant effects [71]. Furthermore, various quercetin concentrations could result in varying terminates considering mitochondrial biogenesis. Davis et al. [63] proposed that applying quercetin at concentrations 12.5 or 25 mg/kg for seven days by gavage sets off mitochondrial biogenesis in mouse muscle and brain by involving the upregulation of cytochrome c, PGC-1*α*, and NAD + -dependent protein deacetylase SIRT1. Additionally, examining 25 mg/kg quercetin (assumed as high dose) stimulates mtDNA levels in brain and muscle tissues. Potentially, modulation of PGC-1*α* by SIRT1 occurs through deacetylation of this coactivator function, amplifying and generating mitochondrial biogenesis by triggering engaged transcription factors in the mechanism [66]. Kim et al. [70] reported an alternative mechanism, whereby quercetin probably stimulated mitochondrial biogenesis. It presented upregulating of Nrf-2/HO-1/PGC-1*α* axis in the liver. The animals were held below a high-fat diet supplied with 0.05%–0.1% quercetin for nine weeks. These authors examined the administrating of 0.01% quercetin for nine weeks, which stimulated the complex IV expression in mice liver supplemented with a high-fat diet. Liu et al. [68] manifested that in rats hippocampus exposed to hypobaric hypoxia; a surge of DNA content, number, length, and mitochondrial area occurs when quercetin (50, 75, or 100 mg/kg/day) is administrated orally for seven days. Consuming 50–100 mg/kg daily quercetin leads to a rise of TFAM, NRF-1, SIRT1, and PGC-1*α* in the rat hippocampus experiencing hypobaric hypoxia in a dose-dependent fashion. On the other hand, the group taking 100 mg/kg quercetin demonstrated a function upregulation of complexes II, IV, and V. Besides, elevated ATP in the hypobaric hypoxia exposed rat hippocampus was observed. The authors further manifested that 50–100 mg/kg quercetin plunged the expression of dynamin-related protein 1 (Drp1), and 75 or 100 mg/kg quercetin plunged fission one homolog protein (Fis1)'s expression. Moreover, 100 mg/kg quercetin upregulated the expression of mitofusin 1 and 2 proteins (Mfn1 and 2). Drp1 and Fis1 are engaged in the regulation of mitochondrial fission [72, 73], and Mfn1 and Mfn2 modulated mitochondrial fusion. Elevated Fis1 results in autophagy [74]. Therefore, quercetin incompletely hampered cellular mitophagy challenging with hypoxia. Overall, similar data propose that quercetin plunges mitochondrial degradation through reducing fission and stimulating fusion. Lately, Sharma et al. [69] investigated that acquiring a daily quercetin regimen for 12 weeks by gavage stimulated the biogenesis of mitochondria in a PGC-1*α*/NRF-1-NRF-2-TFAM-related process in the aluminum-exposed corpus striatum and hippocampus of rats. Moreover, quercetin maintained the mitochondrial shape and elevated mitochondria quantitatively in the brains of aluminum exposed animals. Mitochondrial biogenesis stimulator functions of quercetin were analysed in some investigation models such as exercise in which 25 mg/kg is consumed by gavage during six weeks [75] and conducted high-fat diet studies in which 10 mg/kg is consumed by gavage during five days per week for eight weeks [76] in lab animals. Surprisingly, another investigation [77] discovered that a high-fat diet mouse acquired with 17 mg/kg quercetin for nine weeks generated elevated mitochondrial DNA in skeletal muscles (quadriceps and gastrocnemius) but declined expression of cytochrome c oxidase and NADH dehydrogenase (complex I) subunits. The authors did not analyse if the modulation impacted mitochondrial function as a whole, or these modulations caused the uncoupling of mitochondria and further ATP generation deterioration. Additionally, quercetin failed to spark the biogenesis of mitochondria in the human skeletal muscle [64]. Hence, the role of quercetin as a stimulator agent of mitochondrial biogenesis is vague. Further information demands to comprehend the task, as an example, carbon monoxide and SIRT1 in similar biological events. Moreover, more analyses assist in confirming more certainly whether there is an alternative role in signaling pathways terminating in the enhancement of mitochondrial biogenesis mediated by quercetin. For example, AMPK/protein kinase B, following quercetin's potential generation in declining cellular energy, possibly leads to an elevation in AMP and consequently triggers AMPK/SIRT/PGC-1*α* signaling pathway. Notably, the debates about quercetin and its initiating potential on the biogenesis of mitochondria require to be more illuminated. Therefore, similar biological parameters demonstrate substantial results on human health in disease treatment and prophylaxis in connection with mitochondrial deterioration [78]. In spite of the severity of osteoarthritis (OA), ongoing medical approaches focus on managing symptoms and easing pain, and currently, there is no medication with high treatment efficacy. To discover the therapeutic impact of quercetin on osteoarthritis remission, an OA rat model was experimented through analysing the ECM (extracellular matrix) integrity, ROS quantity, and mitochondria activity. Quercetin could hinder ROS production and upregulate the expression of glutathione and glutathione peroxidase (GSH and GPx) in OA rats. Besides, it showed pivotal effects on oxygen utilization, mitochondrial ATP amount, stimulating mitochondrial membrane potential, and also elevating mitochondrial number. Additionally, quercetin could cease the interleukin-1*β*-induced condensed NO matrix metalloproteinase 3 and 13 (MMP-3 and -13). Thus, the potential therapeutic effect of quercetin on OA was possibly in connection with the AMPK/SIRT1 signaling pathway. In conclusion, quercetin promotes OA treatment by lowering the ROS quantity, conversing with the impairment of mitochondria, and maintaining the extracellular matrix of joint cartilage's integrity, which possibly engage in adjusting of AMPK/SIRT1 signaling pathway [79, 80].

#### 4.1.3. Hydroxytyrosol

**Figure 3:**
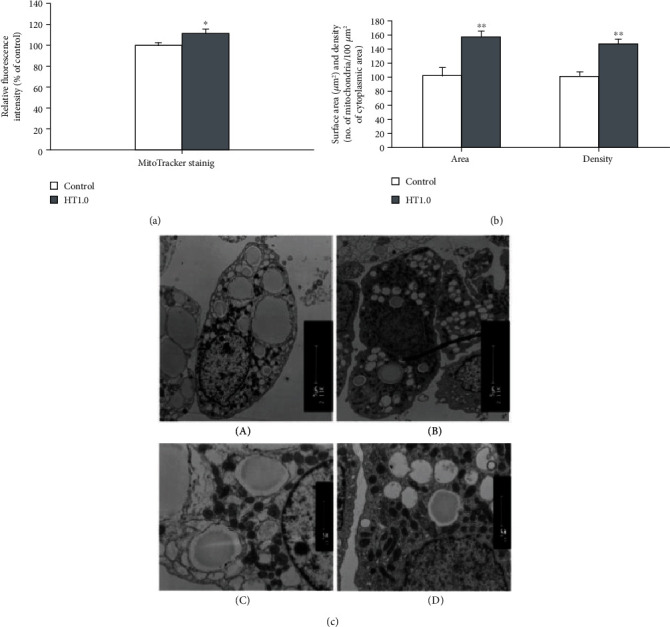
Alteration in ultrastructural and mass of adipocyte mitochondria induced by hydroxytyrosol treatments (1.0 *μ*M for 48 h). (a) Upsurge in the fluorescence intensity in MitoTracker staining. (b) Significant change (*p* < 0.01) in mitochondrial surface area and density in morphometric analysis using electron microscopy. (c) Electron microscope comparison of mitochondrial profiles (A, B) (magnification ×2110) and (C, D) (magnification ×11,000). Reproduced with permission of Elsevier [83].

Hydroxytyrosol as a polyphenol that has strong ties with mitochondrial biogenesis appears in olives and extra virgin olive oils. Conducted *in vitro* studies manifest the potential of hydroxytyrosol in activating PGC-1*α* by deacetylation through SIRT1, which promotes the biogenesis of mitochondria within retinal pigment epithelial cells, ARPE-19 [81]. In the former group's later studies, it has been shown that rats receiving hydroxytyrosol regulate the expression of mitochondrial complexes I and II in skeletal muscle submitted to ergometric exercise and also their PGC-1*α* function. Furthermore, among the trained group, the potential growth of endurance exercise was also determined [82]. Furthermore, although between moderate exercise experimental groups, the enhancement of mitochondrial biogenesis was demonstrated, and extreme exercise reduced the PGC-1*α*. This phenomenon was completely reversed by administration of hydroxytyrosol. Another study manifested the consequences of this polyphenol in improving the function of mitochondria in murine 3T3-L1 adipocytes (Figure 3). It comprised an enhanced function and protein expression of mitochondrial complexes I, II, III, and V, including extended oxygen utilization and declined free fatty acid content [83].

Moreover, collected data from human fibroblasts (*in vitro*) presented the impacts of hydroxytyrosol on the PGC-1*α* cascade function. The stated response is related to surges in phosphorylation of PKA and CREB, engaging processes in coordinating OXPHOS [84]. Hydroxytyrosol, besides stimulating PGC-1*α* expression also within endothelial cells, presents NRF-1 and TFAM stimulation and increases mitochondrial DNA content and ATP synthesis [85].

#### 4.1.4. Other Polyphenols

As well as hydroxytyrosol, resveratrol, and quercetin, there are other polyphenols with stimulating comparable outcomes on mitochondria. The activating of mitochondrial biogenesis through the PGC-1*α*/SIRT1 pathway was determined in rabbit's proximal renal tubular cells when exposed to isoflavones (daidzein, genistein, and formononetin) [86], flavones (wogonin and baicalein) in L6 skeletal muscle cells [87], and flavan-3-ol, in skin fibroblasts from Down's syndrome patients [88]. *In vivo* assessment of green tea's efficacy on the biogenesis of mitochondria in rats indicated a rise in the mtDNA content, including mRNA and proteins of TFAM, PGC-1*α*, and complex IV [89]. Administration of epicatechin-rich cocoa in an experiment of T2D human patients suffering from heart failure enhanced the expressions of SIRT1 and PGC-1*α* and consequently enhanced the biogenesis of mitochondria among skeletal muscle. Besides, it elevated mitochondrial complex I, mitochondrial complex V, and porin [90]. The positive efficacy of long-term acquired treatment of curcumin, a turmeric stemmed polyphenols, and in coordinating the biogenesis of mitochondria has been presented [15, 91]. Curcumin affects the brain of fast-aging augmented senescence-8 mice, develops the PGC-1*α* protein expression, and stimulates mitochondrial membrane potential (MMP) and ATP, along with maintaining the mitochondrial fusion process, pointing its preserving role in diseases resulting from mitochondrial impairment [92]. Following prior investigations, a later study presents that the administration of curcumin elevated the levels of mitochondrial respiratory complexes (exclusively complex IV), PGC-1*α*, and TFAM, which also increased ATP in the APO3-mutant mice's brain [93]. One of South America's most consumed plants is Yerba mate (Ilex paraguariensis). It has abundant bioactive composites as well as polyphenols. It has been shown that increment in mitochondrial spare respiratory capacity and mitochondrial DNA content was observed in cultured muscle C2C12 cells due to yerba mate consumption. In obese mice with high-fat diet, yerba mate enhances mtDNA levels as well as energy outflow in brown adipose tissue and skeletal muscle. Notably, the stated impacts had close bounded to bodyweight decline. Hence, yerba mate could relatively hinder diet-related obesity through escalating ATP consumption and induction of the biogenesis of mitochondria by AMPK/SIRT1/PGC-1*α*-mediated cascade [94]. The green tea's efficacy on the biogenesis of mitochondria was investigated *in vivo*. Epicatechin-rich cocoa administration throughout a study in T2D human patients suffering from heart failure expressed an increase in the SIRT1 and PGC-1*α* expression that promoted mitochondrial biogenesis in skeletal muscle. Furthermore, the amounts of mitochondrial complex I and mitochondrial complex V and porin have also escalated [90]. The favorable impacts of chronic administration of curcumin in mediating mitochondrial biogenesis have also been reported. Curcumin enhanced the PGC-1*α* protein expression in the brain of fast-aging accelerated senescence-8 mice and increased the MMP and ATP contents. Moreover, it replenished the mitochondrial fusion phenomenon, supporting the involvement of mitochondrial dysfunction in disease progression [92]. Another experiment indicated the role of curcumin supplementation in the elevation of TFAM and PGC-1*α* and respiratory chain complexes in vivo in brain tissues [93].

One study has covered the compound of citrate synthase (CS) function in rat skeletal muscle, curcumin therapy on the expression of OXPHOS subunits, AMPK, mtDNA copy number, SIRT1, PGC-1*α*, and endurance training (eTR). Before administering 50 mg/kg Bw/day as low doses of curcumin or 100 mg/kg Bw/day as a high dose of it intraperitoneally through injection in all animals diluting it in dimethyl sulfoxide is a prerequisite. This evaluates curcumin's effect alone and the incorporation effects of curcumin eTR. To determine the proteins' presence, Western blotting and immunoprecipitation were executed. The outcome signified that combined treatment of curcumin with eTR enhanced the expression of CS function and mtDNA copy quantity in the gastrocnemius and soleus muscles as well as elevated SIRT1 expression, PGC-1*α* deacetylation, AMPK phosphorylation, and the ratio of NAD+ to NADH [95]. Epigallocatechin-3-gallate (EGCG) as a mitochondria-aiming molecule stimulates the biogenesis of mitochondria through coordinating chief modulators of mitochondrial metabolism and prohibits mitochondrial dysfunction. Presumably, dietary EGCG declined liver lipid, plasma, and body weight. Moreover, in diet provoking obese mice improved fecal lipid excretion. Such EGCG contributions were moderately mediated through the gene regulation associated with biogenesis of mitochondria and thermogenesis. EGCG could have direct bonds on development of AMPK, triggered in brown adipose tissue and mitochondrial DNA replication. Therefore, EGCG could prohibit obesity (as a moderately linked factor with gene expression regulators of various genes throughout the biogenesis of mitochondria and thermogenesis), AMPK triggering in brown adipose tissue (BAT) of diet-induced obese mice, and stimulate mDNA replication. Stated exhibits propose that EGCG probably performs undeniable tasks in modulating the biogenesis of mitochondria and brown adipose tissue thermogenesis for obesity improvement [96].

Peracetylated (-)-epigallocatechin-3-gallate (AcEGCG) and its 4^″^-O-alkyl congeners catered by formerly exhibited mechanisms were assessed biologically. Notably, EGCG and AcEGCG had trivial efficacy in enhancing the biogenesis of mitochondria. EGCG is recognized to be a mitochondrion-targeting medicinal compound, mediating mitochondrial metabolism, including mitochondrial biogenesis [97]. However, AcEGCG congeners carrying an alkyl group at the 4^″^-O position alteration exhibited a considerable elevation in biological functions when set on their root composite. Among the congeners, 3f with methyl-branched carbonate chain at the 4^″^-O position presented the most stimulation on enhancing mitochondrial biogenesis. 3f treatment received Hepal-6 cells protested increasing 1.5 times in mitochondrial mass and 1.5 times in corresponding mitochondrial DNA content with nDNA. Besides, as a mitochondrial biogenesis stimulator, it elevated mitochondrial activity modulators' expression, comprising 4.2-fold in SIRT1, 1.6-fold in NRF-1, 4-fold in PGC-1*α*, 1.7-fold in NRF-2, 1.6-fold in mtTFA, 1.8-fold in ERR*α*, and 2.5-fold in p-AMPK. An examination's results in the accompany of 3f on oxidative phosphorylation of mitochondria exhibited that 3f enhanced the ratio of NAD+ to NADH, cytochrome c number, ATP synthesis, and oxygen utilization in Hepa1-6 cells by 2.2-, 1.4-, 1.5-, and 2.1-fold, accordingly. Altogether, outcomes proclaim and extent the structure-function association study for EGCG isolation to achieve new stimulators for the biogenesis of mitochondria [98].

A class of polyphenols known as procyanidins is attainable in apples that possess expressed efficacy on the circulatory system and skeletal organs. OA as a locomotive syndrome through a histology perspective is characterized by cartilage diminishment linked to deficiency of proteoglycan homeostasis in chondrocytes. Yet, there is still no effective therapy to preserve diminishing cartilage. The beneficial effects of apple polyphenols (procyanidins) or cartilage homeostasis have been examined. According to the assay's manifestations (in vitro), apple polyphenols enhance the function of mitochondrial dehydrogenases related to escalating mitochondrial DNA copy number and gene expression of PGC-1*α*. Stimulation of proteoglycan biosynthesis through primary chondrocyte upregulation of aggrecan occurs because of procyanidins found in apples. Notice, articular cartilage degradation in OA models in mice responded promising to oral treatment with apple procyanidins and was escalated through chondrocytes' mitochondrial impairment. According to their research, the authors proposed that apple procyanidins are reassuring OA development restrains through stimulating proteoglycan homeostasis and the biogenesis of mitochondria in chondrocytes [99].

### 4.2. Flavonoids

Phosphorylation of AMPK leads to increased levels of mitochondrial biogenesis factors that could be decreased by H2O2. Digitoflavone could have positive effects in this procedure by elevating mitochondrial biogenesis factors besides the regulation of AMPK [100]. *In vitro* studies showed that anthocyanins elevate the expression of mitochondrial TFAM, PGC-1*α*, and NRF-2. These increased levels could affect the phosphorylation of AMPK and mitochondrial biogenesis [101, 102]. Moreover, during brown adipogenesis, anthocyanin decreases oxidative stress and elevates thermogenin (UCP1) expressed in brown fat cells [103, 104].

The function of brown adipocytes depends on the expression of different genes such as protein kinase B (AKT), extracellular signal-related kinases, mitogen-activated protein kinase (ERK-MAPK), NRF-1, and NRF-2. Briefly, elevated mitochondrial amounts of UCP1, cytochrome c (Cytc), PGC-1*α*, and PR16 domain (PRDM16) participate in the lipid metabolism of the body through initializing of AKT and ERK-MAPK [105, 106].

Rutin is a flavonoid that is derived from Saussurea involucrate with proven antifatigue properties. Su et al. showed that the oral administration of rutin to the mice improved the different problems, which related to physical fatigue. The administration of this flavonoid decreased lactic acid levels in the blood and malondialdehyde (MDA) levels in the muscle and brain. Moreover, the administration of rutin regulated SOD and GPx and increased PGC-1*α* and sirtuin 1 (SIRT1) in the muscle and brain that caused elevated levels of maximal endurance capacity. The evaluation of antianxiety effects of rutin on the brain of mice showed regulation of TPI, GDI, and CB1, which are known as anxiety-related proteins [107].

Glycyrrhizic acid (GA), as a major component of *Glycyrrhiza glabra* roots, belongs to flavonoids, which exhibits antioxidant, anti-inflammatory, and antiexcitotoxic properties [108]. GA substantially increases the expression rate and mass of mitochondrial genes subsequent to aluminum toxicity [109]. The protective role of GA against aluminum-induced toxicity was proved to be in tight connection with the improvement of mitochondrial function and biogenesis [109]. Since aluminum toxicity is a good model of neurodegenerative disease, GA could be considered as a supplementary natural product in this context. Moreover, GA has significantly increased the expression of SIRT1 in high glucose-exposed renal tubular epithelial cells [110]. Mitochondrial DNA copy number and mitochondrial transcriptional activity have shown an upward trend in hypoxia/reoxygenation-induced human coronary artery endothelial cell injury after GA treatment [111].

**Figure 4:**
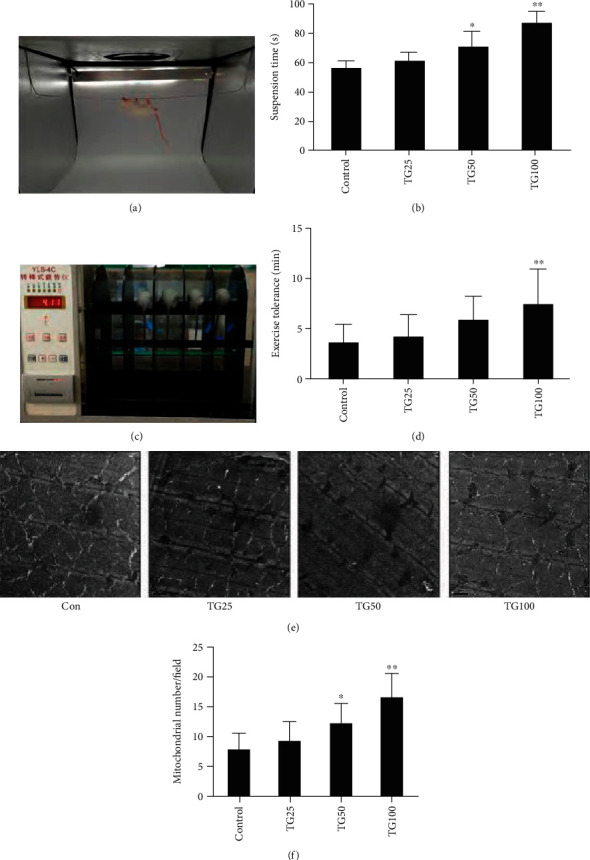
Improvement of exercise performance and skeletal muscle mitochondrial biogenesis of mice using tangeretin. Hanging wire test (a, b), exercise tolerance test (c, d), transmission electron microscope micrographs (e), and mitochondrial numbers (f). Con (control), TG25 (25 mg/kg tangeretin group), TG50 (50 mg/kg tangeretin group), and TG100 (100 mg/kg tangeretin group) (mean ± SD), ^∗^*p* < 0.05, ^∗∗^*p* < 0.01 in comparison with the control group. Scale bars = 1 *μ*m. Reprinted (adapted) with permission from [112]. Copyright (2018) American Chemical Society.

Tangeretin is another flavonoid with positive effects on mitochondrial biogenesis. This substance is extracted from mandarin fruits. The study conducted on Kunming mice, and C2C12 myoblasts for evaluation of mitochondrial biogenesis revealed the significant activation of the signaling pathway of AMPK-PGC1-*α* in C2C12 myoblasts and remarkable improvement on the physical fatigue of mice. Based on these results, it could be suggested that this flavonoid might be a new potential mitochondrial regulator with sufficient results (Figure 4) [112].

Cyanidin-3-glucoside (Cy3g) is another flavonoid, which is founded in different fruits. The effects of this component on mitochondrial biogenesis showed increased levels of intracellular mitochondrial reductase, mitochondrial membrane potential, and ATP production. This substance also upregulates the expression of PGC-1*α* and SIRT1 in a dose- and time-dependent manner. Moreover, the expression of NRF1 and TFAM was increased in the presence of Cy3g. The upregulated amounts of the mentioned genes indicate the improvement of mitochondrial function and biogenesis in presence of Cy3g [113].

Isorhamnetin (ISOR), 3-O-methylquercetin, is a quercetin-derived flavonoid with antiobesity effects. It seems that the antiobesity effects of this metabolite relate to the regulation of mitochondrial biogenesis in adipocytes through the expression of mitochondrial genes, activating AMPK, and replicating of mtDNA [114].

Nobiletin is a natural flavonoid, which is known for anti-inflammatory, anticancer, and antioxidative properties. Dusabimana et al. evaluated the effects of nobiletin (5 mg/kg) on C57BL/6 mice with hepatic ischemia, which was caused by mitochondrial dysfunction and impaired autophagy.

Nobiletin decreases inflammation, oxidative damage, and necrosis by improving mitochondrial biogenesis and functions besides controlling autophagy. The positive effects of nobiletin on mitochondrial biogenesis are related to the regulation of SIRT-1/FOXO3a and PGC-1*α* and AKT pathways as well as other flavonoids [115]. SIRT1 is an important factor for the deacetylation of PGC-1*α*, which is considered as a transcription factor that plays an important role as a regulator in mitochondrial biogenesis and metabolism [113]. Activation of this sensor consequent to cellular stress and caloric restriction leads to the regulation of genes that associate with mitochondrial biogenesis and function. Moreover, reduced levels of FOXO3a due to the deacetylation of this component occurred after the activation of SIRT1 [116–118]. This important metabolic sensor is one of the main targets for most of the flavonoids for regulating metabolic activities and mitochondrial biogenesis [119].

Eriocitrin (eriodictyol 7-rutinoside) is a flavonoid that is found on Citrus limon with notable antiobesity effects. This bioactive substance, while administered orally, enhanced liver function through upregulation of genes that contribute to mitochondrial biogenesis including cytochrome c oxidase subunit 4, TFAM, NRF1, and ATP synthase. Moreover, in HepG2 cells, mitochondrial size and mtDNA amounts were increased in the presence of eriocitrin that could lead to the producing of ATP. Therefore, this could be concluded that intake of eriocitrin in the daily diet activates mitochondrial biogenesis, which improves diet-induced hepatic steatosis [120]. 5-Hydroxy-7-methoxyflavone, 5-hydroxy-3,7,40-trimethoxyflavone, and 5,7-dimethoxyflavone are common flavonoids that are founded in black ginger extract (KPE). KPE showed notable positive results in the treatment of obesity-associated problems and lipid metabolism through activation of brown adipocytes and lipolysis. The effects of these flavonoids on mitochondrial biogenesis and lipid metabolism on C2C12 myoblasts indicate that mitochondrial biogenesis and ATP production improved through regulation of PGC-1*α* and AMPK pathways [121].

Sudachitin is extracted from citrus sudachi fruit. This flavonoid ameliorates metabolic disorders and dyslipidemia through improving mitochondrial biogenesis and triglyceride reduction. This substance elevates the expression levels of SIRT1 and PGC-1*α* as well as nobiletin and other flavonoids, which leads to better mitochondrial biogenesis and metabolic function [122].

## 5. Other Compounds

Saponins, such as tomatine and digitonin, have also been found to be effective compounds in mediating mitochondrial biogenesis. It has been reported that chikusetsu saponin has plummeted the oxidative hazard in neuroblastoma cells which had been exposed to H2O2 through the elevation of PGC1a and SIRT-1 activation. The rhizome of Panax japonicus, which has been shown to possess anti-inflammatory and antioxidant effects, contains chikusetsu saponin [123]. The ethanolic extract of Platycodon grandiflorum has exhibited beneficial effects in brown adipose cells in combating obesity via the upregulation of mitochondrial genes such as PGC1*α*, SIRT3, and Nrf [124]. Amla is another traditional plant with antioxidant and antiaging effects. The protective effects of amla in myotubes subjected to tBHP have been observed in the context of mitochondrial function and biogenesis. The activation of AMPK and Nrf signaling in cells has been attributed to these protective effects [125].

## 6. Conclusion

The efficiency of homeostasis in healthy eukaryotic cells incorporated with mitochondrial biogenesis and function. Physiologically, cells react to diminished energy by either up- or downregulating the transcription factors which stimulate and/or inhibit mitochondrial biogenesis. Any dysfunction in this intracellular pathway could cause metabolic syndrome, neurodegenerative diseases, and cancer. Polyphenols are wide-ranged therapeutic agents that founder in the plant kingdom and have been known for their antioxidant, anticancer, and antiaging properties. As described in the current study, these properties were related to the ability of these functional components to modulate the intracellular pathways of mitochondrial biogenesis. This information suggests that polyphenol components of plants, fruits, and natural extracts could be a promising alternative for preventing or treatment of metabolic and aging-related disorders such as type 2 diabetes, cancer, and neurodegenerative diseases. Also, other natural compounds such as flavonoids and saponins can exert therapeutical effects via interfering with mitochondrial biogenesis-related pathways.

The significance of mitochondria in both healthy and pathologic situations highlights the importance of better understanding the element required in mitochondrial biogenesis and turnover. Data suggest that pharmacological interventions in this context might significantly increase mitochondrial utility. Although there is incipient information on the use of polyphenols as mitochondrial biogenesis inducer, they might serve as fundamental opportunities in developing novel drug classes in mitochondrial involved pathologies.

Despite the aforementioned advantages of polyphenols in this context, more human studies are required to prove their beneficial effects on expanding mitochondrial volume and/or density in order to overcome different diseases. The current knowledge has well demonstrated that protective antioxidant role of polyphenols is at least partially due to their capacity to stimulate mitochondrial biogenesis and improve their function, which elevates mitochondrial efficiency resulting in diminished ROS production. Interestingly, mitochondrial produced ROS are vital for mitochondrial quality control. However, contra-indicatory results are obtained from the outputs of human studies directing at finding an ergogenic effect of polyphenols. In addition, polyphenol compounds possess weak pharmacokinetics properties such as low bioavailability and solubility which demands novel technologies to enhance polyphenol delivery to the target tissue.

## Figures and Tables

**Table 1:** Summary of effects of the different natural agents on mitochondrial biogenesis.

Substance	Model (cell or animals)	Result	References
Resveratrol	C57BL/6NIA mice	Resveratrol increased insulin sensitivity, AMP-activated protein kinase (AMPK), and peroxisome proliferator-activated receptor coactivator 1*α* (PGC-1*α*) receptor activity and improved mitochondrial number. Moreover, resveratrol improved motor function and reduced insulin-like growth factor-1 (IGF-1). These changes led to the improvement of health and survival of high-calorie diet mice. Therefore, resveratrol could be considered as a promising substance for the treatment of obesity-related disorders and diseases of aging.	[38]
Resveratrol	C57Bl/6J mice	The administration of resveratrol protected mice against diet-induced obesity and insulin resistance by improving mitochondrial function and activating SIRT1 and PGC-1*α*.	[39]
Resveratrol	Endothelial cells	The mitochondrial biogenesis of endothelial cells was increased in presence of resveratrol by activating the PGC-1*α*/SIRT1 cascade.	[40]
Resveratrol	Transgenic rats	The administration of resveratrol for transgenic rats enhanced mitochondrial biogenesis and ameliorated Ang II-induced cardiac remodeling.	[41]
Resveratrol	C57BL/6J mice	Resveratrol activated AMPK and increased NAD+ in a SIRT1 dependent manner. This procedure led to the improvement of mitochondrial function.	[46]
Resveratrol	SIRT1 KO mice	Moderate doses of resveratrol improved mitochondrial function by SIRT1, which is required for AMPK activation.	[50]
Resveratrol	HepG2 cells	Resveratrol banded to the subunits of nicotinamide adenine dinucleotide (NADH) dehydrogenase to activate the mitochondrial complex I.	[51]
Resveratrol	C57BL/6J mice	Brain mitochondria of young mice were affected by resveratrol. This substance simulated complex I activity while the respiration rate was not improved.	[52]
Resveratrol	Rat	The results demonstrated that resveratrol inhibited the function of complexes I to III of mitochondrial respiratory chain competing with coenzyme Q.	[53]
Resveratrol	C2C12 myoblasts, C3 cancer cells, and mouse embryonic fibroblasts	Resveratrol treatment improved cellular metabolism and growth and mitochondrial fusion. Moreover, cellular respiratory capacity and the activity of complexes I to IV have shown a surge after resveratrol treatment.	[54]
Resveratrol	Rat	Resveratrol had regulatory effects on the synthesis of ATP and complex V activity.	[55]
Resveratrol	Sprague-Dawley rats	Resveratrol demonstrated inhibitory effects on the enzymatic activity of both rat brain and liver F0F1-ATPase/ATP synthase.	[56]
Resveratrol	Human fibroblasts	The resveratrol treatment excessed primary oxygen intake rates and ATP formation on human fibroblasts derived from the skin of patients.	[57]
Resveratrol	Human skin fibroblasts	Resveratrol improved mitochondrial biogenesis using SIRT1- and AMPK-independent pathways. This improvement involved the estrogen receptor (ER) and estrogen-related receptor alpha (ERR*α*) signaling pathway.	[58]
Resveratrol	Complex I-deficient human fibroblasts	Resveratrol treatment reduced oxidative stress in mitochondrial complex I deficiency using SIRT3. The growth in SIRT3 creativity led to dramatic decreases in ROS level and enhancement of SOD2.	[60]
Resveratrol	Bhas 42 cells	Resveratrol enhanced mitochondrial content by protecting against benzo [a] pyrene-induced bioenergetic dysfunction and ROS generation in neoplastic transformation model.	[61]
Quercetin	ICR mice	The administration of quercetin elevated mitochondrial biogenesis and exercise tolerance in the brain and muscle.	[63]
Quercetin	Young adult male	The administration of quercetin increases mtDNA numbers and improved mitochondrial biogenesis that led to enhanced physical performance.	[64]
Quercetin	HepG2 cells	Administration of quercetin increased the mitochondrial DNA content and biogenesis by activating HO-1 in HepG2 cells.	[65]
Quercetin	Obese mice	Administration of quercetin had protective effects on traumatic brain injury of obese mice by regulation of the NRF-2/HO-1/PGC-1*α* signaling pathway.	[67]
Quercetin	Rat	After induction of hypobaric hypoxia in the rat hippocampus, the administration of quercetin led to increased levels of mitochondrial DNA, TFAM, PGC-1*α*, and NRF-1.	[68]
Quercetin	Rodent	The functions of complexes II, IV, and V were elected after the administration of quercetin. Moreover, this substance improved ATP levels, which could affect the activity of OXPHOS. The provocation of mitochondrial biogenesis in presence of quercetin was occurred due to the activation of the PGC-1*α*/NRF-1-NRF-2-TFAM cascade.	[69]
Quercetin	Male C57BL/6 mice	Quercetin enhanced hepatic mitochondrial oxidative metabolism by inducing heme oxygenase-1 via the Nrf-2 pathway.	[70]
Quercetin	Neuronal cell	Quercetin reduced ischemic neuronal cell death by preserving mitochondrial spare respiratory capacity. Moreover, this substance completely blocked neuroprotection by oxide synthase.	[71]
Quercetin	Wistar rats	The combination of oral quercetin supplementation and exercise prevents brain mitochondrial biogenesis.	[75]
Quercetin	Male C57BL/6 mice	Exercise, but not quercetin, ameliorates inflammation, mitochondrial biogenesis, and lipid metabolism in skeletal muscle after strenuous exercise by high-fat diet mice.	[76]
Quercetin	C57BL/6J mice	Quercetin increased skeletal muscle mitochondrial number and function.	[77]
Quercetin	OA rat model	The administration of quercetin in OA rats reduced ROS levels and ameliorated mitochondrial damages which led to the preservation of the integrity of the extracellular matrix of joint cartilage. This procedure might involve the regulation of the AMPK/SIRT1 signaling pathway.	[79]
Hydroxytyrosol	Retinal pigment epithelial cells	As shown in the retinal pigment epithelial cells, hydroxytyrosol deacetylated through SIRT1 and activated PGC-1*α*, which promoted mitochondrial biogenesis.	[81]
Hydroxytyrosol	Rat	The administration of hydroxytyrosol regulated the expression of mitochondrial complexes I and II in skeletal muscle by the PGC-1*α* signaling pathway.	[82]
Hydroxytyrosol	Murine 3T3-L1 adipocytes	The administration of hydroxytyrosol improved protein expression and function of mitochondrial complexes I, II, III, and V.	[83]
Hydroxytyrosol	Human fibroblasts	Hydroxytyrosol increased the phosphorylation of PKA and CREB, which regulated the biogenesis of OXPHOS.	[84]
Hydroxytyrosol	Endothelial cells	Hydroxytyrosol stimulated PGC-1*α* expression, which led to NRF-1 and TFAM stimulation, the elevation of mitochondrial DNA content, and ATP synthesis.	[85]
Isoflavones (daidzein, genistein, and formononetin)	Rabbits' proximal renal tubular cells	Rabbit's proximal renal tubular cells in exposer to daidzein, genistein, and formononetin had shown elevated mitochondrial biogenesis through the PGC-1*α*/SIRT1 pathway.	[86]
Flavones (wogonin and baicalein)	Rats' L6 skeletal muscle cells	Antimycin A-induced mitochondrial dysfunction of rat L6 cells was ameliorated by Scutellaria baicalensis extracts.	[87]
Flavan-3-ol	Skin fibroblasts from Down's syndrome patients	Epigallocatechin-3-gallate prevents oxidative phosphorylation deficit and promotes mitochondrial biogenesis in human cells from subjects with Down's syndrome.	[88]
Green tea's polyphenols	Rats	Green tea elevated mtDNA contact, mRNA, and proteins of TFAM, PGC-1*α*, and complex IV in mitochondria.	[89]
Epicatechin-rich cocoa	Patients with type 2 diabetes	The expressions of SIRT1 and PGC-1*α* were enhanced in T2D human patients after administration of epicatechin-rich cocoa. This enhancement led to the improvement of mitochondrial biogenesis in skeletal muscle.	[90]
Curcumin	Mice	The mitochondrial membrane potential (MMP) and ATP contents in the brain of fast-aging augmented senescence-8 mice were increased due to the enhancement of PGC-1*α* protein expression in presence of curcumin.	[92]
Curcumin	Mice	The administration of curcumin supplementation elevated the levels of PGC-1*α*, TFAM, ATP, and levels of mitochondrial respiratory complexes in the APO3-mutant mice's brain.	[93]
Yerba mate	Obese mice	C2C12 cells were showed increased mitochondrial respiratory capacity and DNA content in presence of yerba mate. Moreover, in the obese mice, this substance increased mtDNA levels in brown adipose tissue and skeletal muscle. These effects were related to the AMPK/SIRT1/PGC-1*α*-mediated cascade in presence of yerba mate.	[94]
Curcumin	Rat skeletal muscle	The increasing of cAMP levels, mtDNA amounts, SIRT1 expression, PGC-1*α* deacetylation, AMPK phosphorylation, and NAD^+^/NADH ratio was observed due to the curcumin treatment on skeletal muscle.	[95]
Epigallocatechin-3-gallate (EGCG)	Obese mice	EGCG modulated the biogenesis of mitochondrial and brown adipose tissue thermogenesis through AMPK triggering in brown adipose tissue and stimulating the mitochondria DNA replication.	[96]
Epigallocatechin gallate (EGCG)	Hepa1-6 cells	The amounts of the cytochrome C, oxygen consumption, ATP synthesis, and NAD^+^/NADH ratio were increased by modified EGCG derivatives in Hepa1-6 cells.	[98]
Procyanidins	Mice	The expression of the PGC-1*α* gene and copy number of DNA were increased after oral administration of apple procyanidin in OA models of mice.	[99]
Digitoflavone	PC12 cells	Mitochondrial biogenesis improved in presence of digitoflavone by regulation of AMPK and increasing of antioxidant capacity of cells.	[100]
Anthocyanins	3T3-L1 preadipocytes	Anthocyanin inhibited adipocyte differentiation through activation of the AMPK signaling pathway.	[101]
Cyanidin-3-glucoside	3T3-L1 preadipocytes	The intracellular levels of CAMP were signification increased in preadipocytes after Cy36 exposure.	[102]
Anthocyanins	3T3-L1 preadipocytes	The lipogenesis stage during adipocyte differentiation of 3T3-L1 preadipocytes was inhibited by anthocyanins. This substance regulated BAT's function through AKT and ERK signaling pathways.	[103]
Mulberry anthocyanins, cyanidin 3-glucoside, and cyanidin 3-rutinoside,	BAT-cMyc cell	Mulberry anthocyanins, cyanidin 3-glucoside, and cyanidin 3-rutinoside increase the number of mitochondria during brown adipogenesis.	[104]
Mulberry and mulberry wine	C_3_H_10_T_1/2_ mesenchymal stem cell	The number and function of mitochondria were increased during brown adipogenesis by exposure to the mulberry and mulberry wine.	[105]
Rutin	Mice	The administration of rutin decreased the blood levels of lactic acid in the forced swimming mouse model. Moreover, in these animals, the levels of malondialdehyde (MDA) were decreased in the muscle and brain. In these tissues, regulation of SOD and GPx increased PGC-1*α* and sirtuin 1 (SIRT1). The antioxidative effects of this flavonoid were also observed in the brain of mice by regulation of TPI, GDI, and CB1.	[107]
Glycyrrhizic acid	Mice	Glycyrrhizic acid (GA) had neuroprotective effects and increased memory and antioxidant-related enzymes.	[108]
Glycyrrhizic acid	PC12 cells	Mitochondrial function and biogenesis were enhanced against aluminum toxicity of PC12 cells by glycyrrhizic acid treatment.	[109]
Glycyrrhizic acid	Renal tubular epithelial cell	The high glucose-related renal tubular epithelial cell injury was ameliorated by glycyrrhizic acid treatment.	[110]
Glycyrrhizic acid	Human coronary artery endothelial cell	Mitochondria regulation due to the glycyrrhizic acid protective effects ameliorated hypoxia/reoxygenation-induced human coronary artery endothelial cell damage.	[111]
Citrus tangeretin	Kunming mice and C2C12 myoblasts	Mitochondrial biogenesis in skeletal muscle was improved by activation of the AMPK-PGC1-*α* pathway in presence of citrus.	[112]
Cyanidin-3-glucoside	Human hepatocyte cell	The treatment of hepatocyte cell line by cyanidin-3-glucoside showed the upregulation of PGC-1*α* and SIRT 1 expression in a dose- and time-dependent manner. Moreover, the expression of NRF1 and TFAM was increased which led to improvement of function and biogenesis of mitochondria.	[113]
Isorhamnetin	3T3-L1 cells	The expression of mitochondrial genes, activating AMPK, and replicating of mtDNA in presence of isorhamnetin led to antiobesity effects through the regulation of mitochondrial biogenesis.	[114]
Nobiletin	C57BL/6 mice	The activation of SIRT-1/FOXO3a-medicated autophagy and mitochondrial biogenesis in presence of nobiletin ameliorated hepatic ischemia and reperfusion.	[115]
Eriocitrin	HepG2 cells	The oral administration of eriocitrin upregulated the levels of cytochrome c, oxidase subunit 4, TFAM, NRF1, and ATP synthase, which improved liver function in contribution to the mitochondrial biogenesis.	[120]
Polymethoxy flavonoids (black ginger extract)	C2C12 myoblasts	The black ginger's flavonoids including 5-hydroxy-7-methoxyflavone, 5-hydroxy-3,7,40-trimethoxyflavone, and 5,7-dimethoxyflavone enhanced ATP production on C2C12 myoblasts through regulation of PGC-1*α* and AMPK pathway.	[121]
Polymethoxylated flavone (sudachitin)	C57BL/6J mice	Sudachitin improved the SIRT 1 and PGC-1*α*, which caused the amelioration of metabolic disorders and dyslipidemia through improving mitochondrial biogenesis.	[122]
Chikusetsu saponin	Neuroblastoma cells	Chikusetsu saponin has plummeted the oxidative hazard in neuroblastoma cells that had been exposed to H2O2 through the elevation of PGC1a and SIRT-1 activation.	[123]
Platycodon grandiflorum	Brown adipose cells	The ethanolic extract of Platycodon grandiflorum upregulated the mitochondrial genes such as PGC1*α*, SIRT3, and Nrf, which has exhibited beneficial effects in brown adipose.	[124]
Amla	C2C12 myotubes	The protective effects of amla in myotubes subjected to tBHP have been observed in the context of mitochondrial function and biogenesis. The activation of AMPK and Nrf signaling in C2C12 myotubes cells has been attributed to these protective effects.	[125]

## Data Availability

This being a review article, no data was generated during the preparation of this manuscript.

## Abbreviations

ROS: Reactive oxygen species
PGC-1*α*: Proliferator-activated receptor-*γ* coactivator
Nrf-1: Nuclear respiratory factor 1
TFAM: Mitochondrial transcription factor A
AMPK: AMP-activated protein kinase
ERR-*α*: Estrogen-related receptor-*α*
mtIF: Initiation factor
mtEFTu: Elongation factors Tu
mtRF1L: Translational release factor 1-like
mtRRF1: Recycling factors 1
SIRT1: Silent information regulator-1
IMM: Inner mitochondrial membrane
OA: Osteoarthritis
GSH and GPx: Glutathione and glutathione peroxidase
MMP: Mitochondrial membrane potential
EGCG: Epigallocatechin-3-gallate
AcEGCG: Peracetylated (-)-epigallocatechin-3-gallate.
